# Acute diverticulitis in immunocompromised patients: evidence from an international multicenter observational registry (Web-based International Register of Emergency Surgery and Trauma, Wires-T)

**DOI:** 10.1007/s10151-023-02758-6

**Published:** 2023-02-07

**Authors:** Dario Tartaglia, Camilla Cremonini, Elena Annunziata, Fausto Catena, Massimo Sartelli, Andrew W. Kirkpatrick, Serena Musetti, Silvia Strambi, Massimo Chiarugi, Federico Coccolini, the WIRES-T for Acute Diverticulitis Study Group, Francesco Salvetti, Paola Fugazzola, Marco Ceresoli, Fabio Benedetti, Nita Gabriela Elisa, Andrey Litvin, Eftychios Lostoridis, Ali Yasen Yasen Mohamed Ahmed, Dimitrios Manatakis, Ionut Negoi, Orestis Ioannidis, Mustafa Yener Uzunoglu, Joel Noutakdie Tochie, Nicola Cillara, Gia Tomadze, Miklosh Bala, Arda Isik, Vinicius Cordeiro Fonseca, Giovanni Bellanova, Wagih Ghannam, Omer Yalkin, Fernando Hernandez Garcia, Fatih Altintoprak, Dimitar Hadzhiev, Mircea Chirica, Monica Zese, Dimitros Balalis, Yunfeng Cui, Davide Luppi, Luigi Romeo, Andrea Muratore, Elia Giuseppe Lunghi, Yovtcho Yovtchev, Evgeni Dimitrov, Ioannis Nikolopoulos, Maid Omerovic, Maurizio Zizzo, Lara Ugoletti, Gianluca Costa, Rocco Scalzone, Stefano Perrone, Savino Occhionorelli, Matteo Nardi, Francesca Gubbiotti, Ali Muhtaroglu, Rosa Scaramuzzo, Helene Corte, Carlos Yanez, Andee Dzulkarnaen Zakaria, Charalampos Seretis, Roberta Gelmini, Vincenzo Pappalardo, Filippo Paratore, Ruslan Sydorchuk, Francesk Mulita, Yasin Kara, Elena Adelina Toma, Michail Vailas, Maria Sotiropoulou, Mahamad Elbahnasawy, Maria Grazia Sibilla, Gennaro Martines, Beslen Goksoy, Dimitar Hadzhiev, Dario Parini, Claudia Zaghi, Mauro Podda, Aleksey Osipov, Giuseppe Brisinda, Giovanni Gambino, Lali Akhmeteli Krstina Doklestic, Zlatibor Loncar, Dusan Micic, Ivana Lešević, Francesca D’Agostino, Ibrahim Umar Garzali, Yaset Caicedo, Lina Marcela, Paola Andrea Gasca Marin, Konstantinos Perivoliotis, Ioannis Ntentas, Arthur Kuptsov, Sharfuddin Chowdhury, Tapan Patel

**Affiliations:** 1 General and Emergency Surgery Unit, Trauma Center, New Santa Chiara Hospital, University of Pisa, Via Paradisa, Pisa, Italy; 2 Department of Surgery, Bufalini” Hospital, Cesena, Italy; 3 Department of Surgery, Macerata Hospital, Macerata, Italy; 4 General, Acute Care, Abdominal Wall Reconstruction, and Trauma Surgery, Foothills Medical Centre, Calgary, Canada

**Keywords:** Acute diverticulitis, Immunocompromised, Surgery, Medical therapy

## Abstract

**Background:**

Immunocompromised patients with acute diverticulitis are at increased risk of morbidity and mortality. The aim of this study was to compare clinical presentations, types of treatment, and outcomes between immunocompromised and immunocompetent patients with acute diverticulitis.

**Methods:**

We compared the data of patients with acute diverticulitis extracted from the Web-based International Registry of Emergency Surgery and Trauma (WIRES-T) from January 2018 to December 2021. First, two groups were identified: medical therapy (A) and surgical therapy (B). Each group was divided into three subgroups: nonimmunocompromised (grade 0), mildly to moderately (grade 1), and severely immunocompromised (grade 2).

**Results:**

Data from 482 patients were analyzed—229 patients (47.5%) [M:F = 1:1; median age: 60 (24–95) years] in group A and 253 patients (52.5%) [M:F = 1:1; median age: 71 (26–94) years] in group B. There was a significant difference between the two groups in grade distribution: 69.9% versus 38.3% for grade 0, 26.6% versus 51% for grade 1, and 3.5% versus 10.7% for grade 2 (*p* < 0.00001). In group A, severe sepsis (*p* = 0.027) was more common in higher grades of immunodeficiency. Patients with grade 2 needed longer hospitalization (*p* = 0.005). In group B, a similar condition was found in terms of severe sepsis (*p* = 0.002), quick Sequential Organ Failure Assessment score > 2 (*p* = 0.0002), and Mannheim Peritonitis Index (*p* = 0.010). A Hartmann’s procedure is mainly performed in grades 1–2 (*p* < 0.0001). Major complications increased significantly after a Hartmann’s procedure (*p* = 0.047). Mortality was higher in the immunocompromised patients *(p* = 0.002).

**Conclusions:**

Immunocompromised patients with acute diverticulitis present with a more severe clinical picture. When surgery is required, immunocompromised patients mainly undergo a Hartmann’s procedure. Postoperative morbidity and mortality are, however, higher in immunocompromised patients, who also require a longer hospital stay.

## Introduction

Acute diverticulitis is the most common complication of diverticular disease [1]. Symptoms, treatments, and outcomes of this disease in immunocompromised patients differ from those of the general population. Several factors may affect different components of the immune system, resulting in a highly heterogeneous presentation and variable severity of disease [2]. In the literature, there is currently no classification in terms of severity [3]. Existing classifications are based on etiology, distinguishing between congenital and acquired immunodeficiencies, the latter being the most frequent [2, 4–21]. The incidence of acute diverticulitis in immunocompromised individuals is as high as 1% compared with 0.02% in the general population [23, 24]. Any intraabdominal infection in immunocompromised patients tends to have more nuanced symptomatology. Laboratory tests are often normal, and it is common not to observe a leukocytosis [25]. Acute diverticulitis in immunocompromised patients has a higher risk of morbidity and mortality compared with those in the general population [23, 26].

Patients receiving an early diagnosis of uncomplicated diverticulitis may be treated with broad-spectrum intravenous antibiotic therapy [27]. According to several studies, immunocompromised patients have a greater risk of complicated disease and a more aggressive surgical approach to contain the sepsis may be justified [28–32]. However, the literature also suggests that immunocompromised patients undergoing emergency surgery have a worse prognosis, with a mortality rate between 5% and 30%, compared with values of around 5% in the general population [22, 33–36]. The aim of this study was to compare features of the disease at diagnosis, the types of treatment, and the outcomes in immunocompromised and immunocompetent patients with acute diverticulitis undergoing either medical or surgical treatments.

## Materials and methods

### Patient selection

We analyzed the data of patients with acute diverticulitis included in a multicenter international registry [Web-based International Registry of Emergency Surgery and Trauma (WIRES-T)] [37]. The project has been registered at ClinicalTrials.gov (NCT03643718) and includes data from patients worldwide. The study period was January 2018 to December 2021. The study was approved by the local ethics committee (Comitato Etico Area Vasta Nord Ovest Wires-t n. 17,575). Diagnosis of acute diverticulitis was carried out by clinical examination, laboratory tests, and computed tomography (CT) scan showing inflamed diverticula in the left colon and sigmoid with or without signs of complications. In some hospitals, according to the admission policy, patients with lower grades of acute diverticulitis may be treated in medical units. Two groups were identified: medical therapy (group A) and surgical therapy (group B). Each group was then divided into three subgroups based on the degree of immunocompromise: immunocompetent (grade 0), mildly to moderately immunocompromised (grade 1), and severely immunocompromised (grade 2) [3, 5]. A score of 1 was assigned to patients with one or more of the following conditions: aged > 70 years; active malignancy without chemotherapy; rheumatologic disorders (therapy without steroids but with other immunosuppressants); inflammatory bowel disease (therapy without steroids but with other immunosuppressants); diabetes; malnutrition (Nutrition Risk Screening > 3); chronic kidney disease with stages IIIb, IV, and V (according to the glomerular filtration rate); chronic hepatic disease (Child–Pugh class B–C); neurodegenerative disease. A score of 2 was assigned to patients presenting with one or more of the following conditions: leukemia or lymphoma, neutropenia (neutrophil count < 1000/mm^3^), ongoing chemotherapy, transplant (solid organ, bone marrow), high-dose steroids therapy (> 20 mg/day prednisone), or acquired immunodeficiency syndrome (AIDS) (with CD4 + count < 200/mm). All patients who did not have any of the above criteria were assigned a score of 0 (Table 1).

**Table 1:** Grading of immunocompromise

Mildly to moderately immunocompromised (Grade 1)	Severely immunocompromised (Grade 2)
Elderly (> 70 years old) Active malignancy without chemotherapy Rheumatologic disorders (with no high-dose steroid therapy and/or other immunosuppressants) Inflammatory bowel disease (with no high-dose steroid therapy and/or other immunosuppressants) Diabetes Malnutrition (Nutrition Risk Screening > 3) Chronic kidney disease with stages IIIb, IV, and V (according to the glomerular filtration rate) Chronic hepatic disease (Child–Pugh class B–C) Neurodegenerative diseases	Leukemia or lymphoma Neutropenia (Neutrophil count < 1000/mm^3^) Ongoing chemotherapy Transplant (solid organ, bone marrow) High-dose steroid therapy (more than 20 mg/day prednisone) AIDS (with CD4 + count < 200/mm^3^)

*AIDS* acquired immunodeficiency syndrome

### Data analysis

Age, sex, body mass index (BMI), American Society of Anesthesiologists–Physical Status Classification System (ASA), previous episodes of acute diverticulitis, clinical condition at presentation (no signs of sepsis, early sepsis without organ dysfunction, severe sepsis with organ dysfunction, septic shock, unresponsive septic shock—according to the Centers for Medicare and Medicaid Services) [38], quick Sequential Organ Failure Assessment (qSOFA) score 2 + [39], Hinchey’s classification [40], World Society of Emergency Surgery (WSES) classification [41], Mannheim peritonitis index (MPI) [42], time from symptom onset to diagnosis, and time from diagnosis to treatment were collected and analyzed in both groups. In group A, variables related to conservative treatment were also considered: treatment (no antibiotic therapy, antibiotic therapy, or percutaneous drainage), duration of antibiotic therapy, and length of hospital stay. In group B, variables related to surgical treatment and postoperative morbidity were considered: operative treatment (Hartmann’s Procedure, resection and primary anastomosis with protecting stoma, resection and primary anastomosis without protecting stoma, or laparoscopic lavage), operative technique (laparotomic, laparoscopic, or laparoscopic converted to open), need for intensive care, damage control surgery, duration of antibiotic therapy, major postoperative complications (Clavien–Dindo grade > 2) [43], length of stay, and in-hospital mortality.

### Statistical analysis

Quantitative parameters were reported as mean and standard deviation for uniformly distributed data, while nonuniformly distributed data were described as the median and interquartile range (IQR). Qualitative parameters were reported as absolute numbers and percentages. For comparative analysis between the three groups (grades 0, 1, and 2 of immunocompromise), we used the ANOVA test for uniformly distributed quantitative samples and the Kruskal–Wallis *H* test for nonuniformly distributed ones. Regarding categorical qualitative data, Pearson’s Chi-square test and Fisher’s exact test were used, where appropriate. Differences were considered statistically significant where the *p*-value was < 0.05. Statistical analysis was conducted using XLSTAT software (Addinsoft, XLSTAT statistical and data analysis solution. Paris, France. https://www.xlstat.com, 2021).

## Results

Data from 482 patients with acute diverticulitis were analyzed: 229 patients (47.5%) underwent medical therapy [M: F = 1:1; median age: 60 (24–95) years] (Group A), while 253 patients (52.5%) received surgical treatment [M:F = 1:1; median age: 71 (26–94) years] (Group B). In group A, 160 patients presented with grade 0 (69.9%), 61 with grade 1 (26.6%), and eight with grade 2 (3.5%). In group B, 97 patients presented with grade 0 (38.3%), 129 with grade 1 (51%), and 27 with grade 2 (10.7%) (*p* < 0.00001).

The characteristics and clinical presentation of group A are described in Table 2. Mean age was significantly different among subgroups of immunocompromised patients (76.3 ± 10.2 years, 64.6 ± 12.9 years, and 51.8 ± 10.3 years, respectively; *p* < 0.0001). The same was true for female sex distribution [74 (46.25%), 39 (63.9%), and 3 (37.5%), respectively; *p* = 0.046], BMI (25.6 ± 3.5 kg/m^2^, 26.8 ± 3.8 kg/m^2^, and 23.5 ± 3.4 kg/m^2^; *p* = 0.014), ASA score [2 (IQR, 3–1), 3 (IQR, 4–1), and 3 (IQR, 4–2), respectively; *p* < 0.0001]. Severe sepsis with organ dysfunction occurred in 12.5% of severely immunocompromised patients, 4.9% of mildly to moderately immunocompromised patients, and none of the immunocompetent patients (*p* = 0.027). There were no significant differences between the three subgroups regarding qSOFA score > 2. In group A, the distribution according to the Hinchey classification showed no significant differences among the three subgroups (Table 3). No significant differences were found among the subgroups in terms of days between symptom onset and diagnosis and hours between diagnosis and treatment (Table 4). Both immunocompetent and mildly to moderately immunocompromised patients had more frequent percutaneous drainage (4.4% and 6.6%, respectively) than the severely immunocompromised patients (0%) (*p* = 0.035). Patients with grade 2 had a longer period of hospitalization [median value of 8 (IQR, 13–6) days] when compared with grades 1 and 0 [median values of 7 (IQR, 24–2) days and 6 (IQR, 28–1) days, respectively; *p* = 0.005].

**Table 2:** General population characteristics and clinical presentation—Group A (medical therapy)

	Overall *N* = 229	Grade 0 *N* = 160	Grade 1 *N* = 61	Grade 2 *N* = 8	*p*-Value
Age, years, mean (± SD)	58.8 (± 15)	51.8 (± 10.3)	76.3 (± 10.2)	64.6 (± 12.9)	** < *0.0001***
Female sex, *n* (%)	116 (50.7)	74 (46.25)	39 (63.9)	3 (37.5)	***0.046***
BMI, kg/m^2^, mean (± SD)	25.8 (± 3.7)	25.6 (± 3.5)	26.8 (± 3.8)	23.5 (± 3.4)	***0.014***
ASA, median (IQR)	2 (4–1)	2 (3–1)	3 (4–1)	3 (4–2)	** < *0.0001***
Previous episodes of acute diverticulitis, *n* (%)	73/227 (32.2)	53/159 (33.3)	15/60 (25)	5 (62.5)	0.097
Clinical condition at presentation, *n* (%)					
No sign of sepsis	100/228 (43.9)	69/159 (43.4)	27 (44.3)	4 (50)	***0.027***
Early sepsis without organ dysfunction	124/228 (54.4)	90/159 (56.6)	31 (50.8)	3 (37.5)	
Severe sepsis with organ dysfunction	4/228 (1.7)	0/159 (0)	3 (4.9)	1 (12.5)	
Septic shock	0/228 (0)	0/159 (0)	0 (0)	0 (0)	
Unresponsive septic shock	0/228 (0)	0/159 (0)	0 (0)	0 (0)	
Quick SOFA score 2 + , *n* (%)	4 (1.75)	2 (1.25)	2 (3.3)	0 (0)	0.547

In bold, *p*-value result is significant (*p* < 0.05)

*BMI* body mass index, *ASA* American Society of Anesthesiologists, *SOFA* sequential organ failure assessment

**Table 3:** Severity of disease—Group A (medical therapy)

	Overall *N* = 229	Grade 0 *N* = 160	Grade 1 *N* = 61	Grade 2 *N* = 8	*p*-Value
Hinchey grade, *n* (%)					
Ia	135 (58.9)	94 (58.75)	35 (57.4)	6 (75)	0.36
Ib	59 (25.8)	46 (28.75)	12 (19.7)	1 (12.5)	
IIa	19 (8.3)	11 (6.9)	8 (13.1)	0 (0)	
IIb	16 (7)	9 (5.6)	6 (9.8)	1 (12.5)	
III	0 (0)	0 (0)	0 (0)	0 (0)	
IV	0 (0)	0 (0)	0 (0)	0 (0)	
WSES grade, *n* (%)					
Uncomplicated	107 (46.7)	72 (45)	30 (49.2)	5 (62.5)	***0.034***
1a	55 (24)	46 (28.8)	9 (14.8)	0 (0)	
1b	32 (14)	20 (12.5)	11 (18)	1 (12.5)	
2a	14 (6.1)	8 (5)	6 (9.8)	0 (0)	
2b	7 (3.1)	4 (2.5)	2 (3.3)	1 (12.5)	
3	12 (5.2)	10 (6.2)	2 (3.3)	0 (0)	
4	2 (0.9)	0 (0)	1 (1.6)	1 (12.5)	

In bold, *p*-value result is significant (*p* < 0.05)

*WSES* World Society of Emergency Surgery

**Table 4:** Outcomes—Group A (medical therapy)

	Overall *N* = 229	Grade 0 *N* = 160	Grade 1 *N* = 61	Grade 2 *N* = 8	*p*-Value
Time since symptoms begin, days, mean (± SD)	3.2 (± 3)	3.3 (± 3.3)	3 (± 2.1)	2.3 (± 2.1)	0.615
Time from diagnosis to treatment, hours, mean (± SD)	6.4 (± 23)	5.7 (± 19)	8.8 (± 32.5)	3.3 (± 4.1)	0.65
Conservative treatment, *n* (%)					
No antibiotic	3 (1.3)	0 (0)	2 (3.3)	1 (12.5)	***0.035***
Antibiotic	215 (93.9)	153 (95.6)	55 (90.1)	7 (87.5)	
Percutaneous drainage	11 (4.8)	7 (4.4)	4 (6.6)	0 (0)	
Duration of antimicrobial therapy, days, median (IQR)	6.5 (24–1)	6 (18–1)	7 (24–2)	8 (13–6)	0.114
Length of stay, days, median (IQR)	6 (28–1)	6 (28–1)	7 (24–2)	8 (13–6)	***0.005***

In bold, *p*-value result is significant (*p* < 0.05)

The characteristics of the general population and clinical presentation of group B are described in Table 5. Sixty patients (24%) needed operative treatment after failure of the medical therapy: 27% of them were treated with percutaneous drainage and 45% were immunocompromised. The mean age was 54.5 ± 10 years in grade 0, 76.4 ± 9.6 years in grade 1, and 71.6 ± 12 years in grade 2 (*p* < 0.0001). Female sex was mainly represented in grade 1 [78 (60.5%)] and grade 2 [16 (59.3%)] (*p* = 0.047). No significant differences were found among the three subgroups with regard to mean BMI. The median ASA value was 2 (IQR, 4–1) in grade 0, 3 (IQR, 4–1) in grade 1, and 3 (IQR, 4–2) in grade 2 (*p* < 0.0001). Previous episodes of acute diverticulitis were more frequent in grade 0 (44.3%), than grade 1 (27.1%) and grade 2 (14.8%) (*p* = 0.003). According to the grade of immunocompromise, excluding those patients who had had previous episodes of acute diverticulitis (overall, *N* = 155), we found that 40/161 (24.8%) of grade 0, 82/140 (58.5%) of grade 1, and 23/26 (88.4%) of grade 2 required emergency surgery during the first hospitalization for diverticulitis. Severe sepsis with organ dysfunction occurred in 10.3% of grade 0, 25.6% of grade 1, and 18.5% of grade 2 (*p* = 0.002). A qSOFA score > 2 was observed in 4.1% of grade 0, 22.5% of grade 1, and 7.4% of grade 2 (*p* = 0.0002). Immunocompetent patients developed localized peritonitis more frequently (40.6%). In contrast, immunocompromised patients developed diffuse peritonitis more frequently (*p* = 0.001) (Table 6). Higher Hinchey grades were found in immunocompromised patients, while the distribution of the WSES classification was not significantly different among the subgroups (Table 6). The mean MPI increased significantly with the severity of immunocompromise (*p* = 0.010). Hartmann’s procedure and resection with primary anastomosis were performed in 7.5% versus 82.8% of grade 0, 44.8% versus 50.4% of grade 1, and 65.2% versus 21.8% of grade 2 (*p* < 0.0001) (Table 7). The open approach was preferred in 73.6% of grade 1, 70.4% of grade 2, and 41.2% of grade 0 (*p* < 0.0001). More frequently, immunocompromised patients required intensive care (10.3% grade 0, 30.2% grade 1, and 29.6% grade 2) (*p* = 0.001). There were no significant differences between the three subgroups concerning the days since onset of symptoms. The mean time from diagnosis to treatment was significantly shorter in grade 2 (8.3 ± 10.6 h versus 63.3 ± 107.6 h in grade 1 versus 98.2 ± 145.7 h in grade 0) (*p* = 0.002). Postoperative complications were more frequently observed in mildly to moderately and severely immunocompromised patients (*p* = 0.0004) (Table 8). Major postoperative complications occurred more frequently in the Hartmann’s procedure group with increasing degree of immunocompromise (*p* = 0.047); this trend was not observed in the other surgical procedures (Table 9). In-hospital mortality was 1% in immunocompetent patients, 10.1% in mildly to moderately immunocompromised patients, and 19.2% in severely immunocompromised patients (*p* = 0.002) (Table 8).

**Table 5:** General population characteristics and clinical presentation—Group B (surgical therapy)

	Overall *N* = 253	Grade 0 *N* = 97	Grade 1 *N* = 129	Grade 2 *N* = 27	*p*-Value
Age, years, mean (± SD)	67.5 (± 14.4)	54.5 (± 10)	76.4 (± 9.6)	71.6 (± 12)	** < *0.0001***
Female sex, *n* (%)	137 (54.15)	43 (44.3)	78 (60.5)	16 (59.3)	***0.047***
BMI, kg/m^2^, mean (± SD)	26 (± 4.3)	26 (± 3.8)	26 (± 4.6)	26 (± 4.2)	0.896
ASA class, median (IQR)	3 (4–1)	2 (4–1)	3 (4–1)	3 (4–2)	** < *0.0001***
Previous episodes of acute diverticulitis, *n* (%)	82 (32.4)	43 (44.3)	35 (27.1)	4 (14.8)	***0.003***
Clinical condition at presentation, *n* (%)					
No sign of sepsis	53 (20.9)	26 (26.8)	25 (19.4)	2 (7.4)	***0.002***
Early sepsis without organ dysfunction	148 (58.5)	61 (62.9)	68 (52.7)	19 (70.4)	
Severe sepsis with organ dysfunction	48 (19)	10 (10.3)	33 (25.6)	5 (18.5)	
Septic shock	3 (1.2)	0 (0)	3 (2.3)	0 (0)	
Unresponsive septic shock	1 (0.4)	0 (0)	0 (0)	1 (3.7)	
Quick SOFA score 2 + , *n* (%)	35 (13.8)	4 (4.1)	29 (22.5)	2 (7.4)	***0.0002***

In bold, *p*-value result is significant (*p* < 0.05)

*BMI* body mass index, *ASA* American Society of Anesthesiologists, *SOFA* sequential organ failure assessment

**Table 6:** Severity of disease—Group B (surgical therapy)

	Overall *N* = 253	Grade 0 *N* = 97	Grade 1 *N* = 129	Grade 2 *N* = 27	*p* value
Peritonitis, *n* (%)					
No sign of peritonitis	56/252 (22.2)	27/96 (28.1)	24 (18.6)	5 (18.5)	***0.001***
Localized	89/252 (35.3)	39/96 (40.6)	48 (37.2)	2 (7.4)	
Diffused	107/252 (42.5)	30/96 (31.3)	57 (44.2)	20 (74.1)	
Hinchey grade, *n* (%)					
Ia	37 (14.6)	15 (15.5)	20 (15.5)	2 (7.4)	***0.036***
Ib	27 (10.7)	15 (15.5)	11 (8.5)	1 (3.7)	
IIa	20 (7.9)	8 (8.2)	9 (7)	3 (11.1)	
IIb	38 (15)	14 (14.4)	23 (17.8)	1 (3.7)	
III	90 (35.6)	37 (38.1)	38 (29.5)	15 (55.6)	
IV	41 (16.2)	8 (8.3)	28 (21.7)	5 (18.5)	
WSES grade, *n* (%)					
Uncomplicated	30 (11.9)	17 (17.5)	13 (10.1)	0 (0)	0.086
1a	17 (6.7)	10 (10.3)	6 (4.6)	1 (3.7)	
1b	24 (9.5)	9 (9.3)	13 (10.1)	2 (7.4)	
2a	37 (14.6)	14 (14.4)	20 (15.5)	3 (11.1)	
2b	28 (11.1)	10 (10.3)	17 (13.2)	1 (3.7)	
3	29 (11.5)	11 (11.3)	12 (9.3)	6 (22.2)	
4	88 (34.8)	26 (26.8)	48 (37.2)	14 (51.9)	
Mannheim peritonitis index, mean (± SD)	13.9 (± 10.5)	10.6 (± 9.2)	15.9 (± 11.1)	16.6 (± 9)	***0.010***

In bold, *p*-value result is significant (*p* < 0.05)

*WSES* World Society of Emergency Surgery

**Table 7:** Operative and perioperative parameters—Group B (surgical therapy)

	Overall *N* = 253	Grade 0 *N* = 97	Grade 1 *N* = 129	Grade 2 *N* = 27	*p*-Value
Time since symptoms begin, days, mean (± SD)	5.6 (± 12.1)	6.1 (± 11.4)	5.8 (± 13.5)	2.6 (± 4)	0.397
Time from diagnosis to treatment, hours, mean (± SD)	70.9 (± 121.3)	98.2 (± 145.7)	63.3 (± 107.6)	8.3 (± 10.6)	***0.002***
Surgical technique, *n* (%)					
Open	154 (60.9)	40 (41.2)	95 (73.6)	19 (70.4)	** < *0.0001***
Laparoscopy	58 (22.9)	34 (35.1)	17 (13.2)	7 (25.9)	
Laparoscopy converted to open	41 (16.2)	23 (23.7)	17 (13.2)	1 (3.7)	
Surgical treatment, *n* (%)					
Hartmann’s procedure	78/241 (32.4)	7/93 (7.5)	56/125 (44.8)	15/23 (65.2)	** < *0.0001***
Primary anastomosis with protecting stoma	41/241 (17)	22/93 (23.7)	18/125 (14.4)	1/23 (4.4)	
Primary anastomosis without protecting stoma	104/241 (43.1)	55/93 (59.1)	45/125 (36)	4/23 (17.4)	
Laparoscopic lavage	18/241 (7.5)	9/93 (9.7)	6/125 (4.8)	3/23 (13)	
ICU admission, *n* (%)	57 (22.5)	10 (10.3)	39 (30.2)	8 (29.6)	***0.001***
Damage control surgery, *n* (%)	16 (6.3)	4 (4.1)	10 (7.75)	2 (7.4)	0.525
Duration of antimicrobial therapy, days, median (IQR)	11 (70–1)	10 (70–3)	12 (58–1)	10 (36–2)	0.326
Length of stay, days, median (IQR)	12 (70–1)	11 (70–1)	13 (58–2)	13 (48–2)	0.315

In bold, *p*-value result is significant (*p* < 0.05)

*ICU* intensive care unit

**Table 8:** Postoperative complications and mortality—Group B (surgical therapy)

	Overall *N* = 253	Grade 0 *N* = 97	Grade 1 *N* = 129	Grade 2 *N* = 27	*p*-Value
Clavien–Dindo grade, *n* (%)					
0	116/252 (46)	61 (62.9)	49 (38)	6/26 (23.1)	***0.0004***
I	36/252 (14.3)	14 (14.4)	17 (13.2)	5/26 (19.2)	
II	49/252 (19.5)	12 (12.4)	32 (24.8)	5/26 (19.2)	
III	27/252 (10.7)	8 (8.3)	14 (10.8)	5/26 (19.2)	
IV	6/252 (2.4)	1 (1)	5 (3.9)	0/26 (0)	
V	18/252 (7.1)	1 (1)	12 (9.3)	5/26 (19.2)	
In-hospital mortality, *n* (%)	19/252 (7.5)	1 (1)	13 (10.1)	5/26 (19.2)	***0.002***

In bold, *p*-value result is significant (*p* < 0.05)

**Table 9:** Major complications: Clavien–Dindo grade (3 +)—Group B (surgical therapy)

	Overall *N* = 51/253	Grade 0 *N* = 10/97	Grade 1 *N* = 31/129	Grade 2 *N* = 10/27	*p* value
Surgical treatment, *n* (%)					
Hartmann’s procedure	30/50 (60)	2/9 (22.2)	21 (67.7)	7 (70)	***0.047***
RPA with protecting stoma	6/50 (12)	3/9 (33.3)	3 (9.7)	0 (0)	0.058
RPA without protecting stoma	8/50 (16)	3/9 (33.3)	4 (12.9)	1 (10)	0.365
Laparoscopic lavage	6/50 (12)	1/9 (11.1)	3 (9.7)	2 (20)	0.822

In bold, *p*-value result is significant (*p* < 0.05)

*RPA* resection with primary anastomosis

## Discussion

The present study compared the clinical presentations, the severity of disease at diagnosis, the types of treatment, and the outcomes between immunocompromised and immunocompetent patients with acute diverticulitis undergoing medical and surgical treatments. To date, the data available in the literature on acute diverticulitis in immunocompromised patients is scarce. The term “immunocompromised” is still not clearly defined [2, 3]. Numerous factors can lead to an impairment of host immune defenses, with heterogeneous phenotypes and different severities. Therefore, we defined two levels of severity: mildly to moderately immunocompromised (grade 1) and severely immunocompromised (grade 2). Most studies define a single category of immunocompromised patients [28, 44–46]. Greenberg et al. identified two types of immunocompromised patients, depending on whether the underlying cause is permanent (AIDS, hematologic malignancies, and intrinsic immune system disorders) or removable (solid malignancies, organ transplantation, and rheumatologic/inflammatory disorders when receiving chemotherapy, immunosuppressants, or corticosteroid therapy) [5].

In this study, we confirm that immunocompromised patients tend to present at diagnosis with more advanced septic conditions; this occurs in both patients treated with medical therapy and those undergoing surgery. Most of the data in the literature comes from studies conducted in groups of transplant recipients or patients undergoing chronic corticosteroid therapy [23, 24, 31]. Patients with immune deficiency developing an episode of acute diverticulitis are likely to be unable to generate an adequate immune response to the infection. This results in a delayed diagnosis, with consequent progression and worsening of the disease. In this study, immunocompromised patients treated with medical therapy had a higher percentage of uncomplicated acute diverticulitis. This could be related to the fact that immunocompromised patients with the complicated disease are more likely to be treated with surgery. Several studies have confirmed that uncomplicated disease detected on CT scan can be successfully treated with antibiotic therapy, even in immunocompromised subjects, without the need for emergency surgery [23, 24, 28]. The efficacy of medical management of diverticular disease basically depends on three factors: severity of diverticulitis, state of patient’s immunocompromise, and adequate use of antibiotics. In the literature, a 20% failure rate of medical therapy for in-hospital patients has been reported [47], which is consistent with our results (60/289, 20.7%). We are unable to establish if the degree of immunocompromise influences the success of conservative therapy, but clearly this must be taken into account before initiating medical management.

Immunocompromised patients treated surgically showed a greater tendency to develop more diffuse peritonitis. This reflected in a higher prevalence of Hinchey grades III and IV and higher MPI values. Biondo et al., in 2012, reported higher rates of peritonitis in immunodepressed patients who underwent surgery [28]. Another study analyzed the postoperative outcomes of solid organ transplant patients who developed an episode of acute diverticulitis—more than half of the subjects developed generalized peritonitis [34]. Consequently, a Hartmann’s procedure is the predominant operative treatment in this group, performed in 65.2% of patients with severe immunocompromise and 44.8% of patients who are mildly to moderately immunocompromised. Others confirm the procedure mostly performed in immunocompromised subjects is a Hartmann’s procedure [28, 35, 48–51]. In contrast, resection with primary anastomosis (RPA) with or without protecting stoma was mainly (82.8%) performed in the group of immunocompetent patients, again consistent with data reported in the literature [30, 48, 52, 53].

Despite the adoption of a more aggressive surgical approach in patients with a compromised immune response, postoperative complications and in-hospital mortality in this population remained high. Patients undergoing a Hartmann’s procedure in our series suffered a higher rate of major complications (Clavien–Dindo grade 3 +); Complications were higher in subjects with severe (70%) and mild-to-moderate (67.7%) immunocompromise than in immunocompetent patients (22.2%). A similar comparison by Biondo et al., indicated a Hartmann’s procedure was performed in 79.2% of immunocompromised patients and only in 23.8% of immunocompetent patients; the high postoperative mortality (28.1%) observed in subjects undergoing a Hartmann’s procedure was attributed by the authors to patient selection rather than to the surgical technique itself [44]. Our observed in-hospital mortality rates are also consistent with the literature, with values of 19.2% in patients with severe immunodeficiency, 10.1% in patients who are mildly to moderately immunocompromised, and 1% in the immunocompetent patients. Studies report wide variability in the postoperative mortality rates of immunocompromised patients, with values ranging from 5% to 30% [22, 23, 26]. Hwang et al. reported postoperative mortality rate of 23% in transplant recipients or in patients on chronic corticosteroid therapy with acute diverticulitis [23]. In contrast, the postoperative mortality rate of acute diverticulitis in the general population ranges from 4.3 to 5.7% [33, 35].

This study has some limitations. The current registry does not guarantee inclusion of all patients: therefore, there was a low contribution of patients for some centers during the study period. This may have led to an underestimation of the true numbers of patients with the disease. Some participating hospitals have a policy of admitting patients with lower grades of acute diverticulitis into medical units, leading to a potential for missing data. As there is neither a clear definition of the term “immunocompromised” nor classification in degrees of severity, the current literature consists of heterogeneous groupings that are subject to the personal interpretations of the authors. In the current study, there is no follow-up data meaning we are unable to estimate the recurrence rate and the need for emergency surgery due to recurrence in immunocompromised patients treated with medical therapy. Finally, the severely immunocompromised patients both in group A (medical therapy) and group B (surgical therapy) are small. One strength of our data is the detailed data regarding the complete course of patients from their admission to the hospital to their discharge. Another strength lies in the possibility of analyzing the same variables in immunocompetent and immunocompromised patients allowing a direct and robust comparison between the two categories.

## Conclusions

Immunocompromised patients with acute diverticulitis tend to present with more advanced disease at diagnosis than immunocompetent patients. This occurs both in patients treated with medical therapy and in those undergoing surgery. Immunocompromised patients receiving medical therapy tend to have uncomplicated disease. Those managed with surgery present with more severe disease, with higher percentages of Hinchey grades III and IV. In the case of surgery, immunocompromised patients undergo Hartmann’s procedure more frequently; in immunocompetent patients, resection with primary anastomosis is more common. Although the conservative approach of resection with end colostomy (Hartmann’s procedure) is the overwhelming surgical choice for the high-risk immunocompromised patient, postoperative morbidity and mortality remain high.

## Data Availability

The datasets generated during and/or analyzed during the current study are available from the corresponding author on reasonable request.

## Appendix

Collaborators’ affiliations.

(1) General Surgery Dept. Pavia University Hospital, Pavia, Italy.

(2) General Surgery Dept., Monza University Hospital, Monza, Italy.

(3) General Surgery Dept., Sant’Anna Hospital, Castelnuovo dei Monti, Italy.

(4) General Surgery Dept., Immanuel Kant Baltic Federal University, Regional Clinical Hospital, Kaliningrad, Russia.

(5) 1st Department of Surgery, Kavala General Hospital, Kavala, Greece.

(6) General Surgery Dept. Mahayil General Hospital, Mahayil, Saudi Arabia.

(7) General Surgery Dept., Athens Naval and Veterans Hospital, Athens, Greece.

(8) General Surgery Dept., Emergency Hospital of Bucharest, Bucharest, Romania.

(9) General Surgery Dept., General Hospital "G. Papanikolaou", Thessaloniki, Greece.

(10) General Surgery Dept., Kestel State Hospital, Bursa, Turkey.

(11) General Surgery Dept., Yaounde Central Hospital, Yaounde, Cameroon.

(12) General Surgery Dept., Santissima Trinità Hospital, Cagliari, Italy.

(13) General Surgery Dept. Tbilisi University Hospital, Tbilisi, Georgia.

(14) General Surgery Dept., Hadassah Medical Center, Jerusalem, Israel.

(15) General Surgery Dept. Erzincan University Hospital, Erzincan, Turkey.

(16) General Surgery Dept. Hospital ViValle, São José dos Campos, Brazil.

(17) General Surgery Dept. Taranto Hospital, Taranto, Italy.

(18) General Surgery Dept. Gizan armed forces hospital, Abu Aresh, Saudi Arabia.

(19) General Surgery Dept., Bursa Ali Osman Sönmez Oncology Hospital, Bursa, Turkey.

(20) General Surgery Dept., Hospital central military, Mexico City, Mexico.

(21) General Surgery Dept., Sakarya University School of Medicine, Sakarya, Turkey.

(22) General Surgery Dept., Plovdiv University Hospital, Plovdiv, Bulgaria.

(23) General Surgery Dept., Grenoble University Hospital, Grenoble, France.

(24) General Surgery Dept., Ferrara University Hospital, Ferrara, Italy.

(25) General Surgery Dept., Saint Savvas Anticancer Hospital, Athens, Greece.

(26) General Surgery Dept., Tianjin Nankai Hospital, Nankai Clinical School of Medicine, Tianjin Medical University, Tianjin, China.

(27) General Surgery Dept., S. Maria Nuova Hospital, Reggio Emilia, Italy.

(28) General Surgery Dept., Sant’Anna Hospital, Ferrara, Italy.

(29) General Surgery Dept., Agnelli Hospital, Pinerolo, Italy.

(30) General Surgery Dept., University Hospital "Prof. Dr Stoyan Kirkovich", Stara Zagora, Bulgaria.

(31) General Surgery Dept., Lewisham & Greenwich NHS Trust, London, United Kingdom.

(32) General Surgery Dept., UKC Tuzla, Tuzla, Bosnia and Herzegovina.

(33) General Surgery Dept., Azienda Unità Sanitaria Locale-IRCCS, Reggio Emilia, Italy.

(34) General Surgery Dept., Azienda Unità Sanitaria Locale, Guastalla, Italy.

(35) General Surgery Dept., Azienda Ospedaliera Universitaria Sant' Andrea Sapienza Università, Roma, Italy.

(36) General Surgery Dept., Sant’Anna Hospital, Castelnuovo dei Monti, Italy.

(37) General Surgery Dept., Monza University Hospital, Monza, Italy.

(38) General Surgery Dept., Sant’Anna Hospital, Ferrara, Italy.

(39) General Surgery Dept., San Camillo Hospital, Roma, Italy.

(40) General Surgery Dept., Bufalini Hospital, Cesena, Italy.

(41) General Surgery Dept., Sakarya Training and Research Hospital, Sakarya, Turkey.

(42) General Surgery Dept., San Donato Hospital, Milano, Italy.

(43) General Surgery Dept., Hôpital Saint-Louis, Paris, France.

(44) General Surgery Dept., Royo Villanova Hospital, Zaragoza, Spain.

(45) General Surgery Dept., Hospital University Sains Malaysia, Kelantan, Malaysia.

(46) General Surgery Dept., George Eliot Hospital NHS Trust, Nuneaton, West Midlands.

(47) General Surgery Dept., Modena University Hospital, Modena, Italy.

(48) General Surgery Dept., Varese University Hospital, Varese, Italy.

(49) General Surgery Dept., Santa Maria delle Croci Hospital, Ravenna, Italy.

(50) General Surgery Dept., Bukovinian State Medical University, Chernivtsi, Ukraine.

(51) General Surgery Dept., Patras University Hospital, Patras, Greece.

(52) General Surgery Dept., Health Sciences University Kanuni Sultan Süleyman Training and Research Hospital, Istanbul, Turkey.

(53) General Surgery Dept., Elias Emergency University Hospital, Bucharest, Romania.

54) General Surgery Dept., Laiko General Hospital, Athens, Greece.

(55) General Surgery Dept., Evagelismos General Hospital, Athens, Greece.

(56) General Surgery Dept., Tanta University Hospital, Tanta, Egypt.

(57) General Surgery Dept., Ferrara University Hospital Ferrara, Italy.

58) General Surgery Dept., Bari University Hospital, Bari, Italy.

(59) General Surgery Dept., Sehit Prof. Dr. Ilhan Varank Training and Research Hospital, Istanbul, Turkey.

(60) General Surgery Dept., University Hospital "Saint George", Plovdiv,Bulgaria.

(61) General Surgery Dept., Rovigo Hospital, Rovigo, Italy.

(62) General Surgery Dept., Vicenza Hospital, Vicenza, Italy.

(63) General Surgery Dept., Cagliari University Hospital, Cagliari, Italy.

(64) General Surgery dept., Scientific research institute of Emergency Medicine, Saint Petersburg, Russia.

(65) General Surgery dept., Fondazione Policlinico Universitario Gemelli, Rome, Italy.

(66) UOC Chirurgia Generale, PO Sant’Antonio Abate, Trapani, Italy.

(67) Tbilisi State Medical University Clinic, Tbilisi, Georgia.

(68) Clinic for emergency surgery, Emergency centre, University Clinical Centre of Serbia, Belgrade, Serbia.

(69) General Surgery Dept., Santissima Trinità Hospital, Italy.

(70) General Surgery Dept., Aminu Kano Teaching Hospital, Nigeria.

(71) General Surgery Dept., Fundacion Valle del Lili, Cali, Colombia.

(72) General Surgery Dept., General Hospital of Volos, Volos, Greece.

(73) Federal State Budgetary Institution City Clinical Hospital named after S.S. Yudin of the Moscow Department of Health, Moscow, Russia.

(74) General Surgery Dept., King Saud Medical City, King Saud City, Saudi Arabia.

(75) Baroda Medical College and SSG Hospital, Baroda, India.
